# Integrated Soil Amendments Alleviate Subsoil Acidification and Enhance Ponkan Seedling Growth in a Column Experiment

**DOI:** 10.3390/plants14233613

**Published:** 2025-11-26

**Authors:** Jiacheng Zhang, Xiaoya Duan, Pengxiao Sun, Fei Zheng, Xiaochuan Ma, Yuan Yu, Yan Li, Ping Wang

**Affiliations:** Key Laboratory of Ministry of Education for Genetics, Breeding and Multiple Utilization of Crops, Institute of Genetics and Breeding in Horticultural Plants, College of Horticulture, Fujian Agriculture and Forestry University, Fuzhou 350002, China

**Keywords:** soil acidification, aluminum toxicity, soil amendments, deep soil, ponkan, growth, photosynthesis, carbon and nitrogen metabolism

## Abstract

The ponkan (*Citrus reticulata* Blanco cv. Ponkan), an important citrus crop, is increasingly threatened by soil acidification. This study evaluated the efficacy of various soil amendments, including lime alone (L), lime with gypsum and organic fertilizer (LGOF), lime plus K_2_CO_3_ (LK), and lime with chicken manure ash (LCMA), in mitigating soil acidification and improving ponkan seedling growth. Surface-applied lime raised topsoil pH and acid buffering capacity while reducing exchangeable Al. However, combined amendments (LGOF, LK, LCMA) more effectively alleviated acidity throughout the soil profile. They significantly increased pH and buffering capacity, decreased exchangeable H and Al in the 20–40 cm layer, and elevated exchangeable base cations (K^+^, Ca^2+^, Mg^2+^). These changes reduced Al content in roots, stems, and leaves, promoted deeper root growth, and increased biomass and nutrient uptake (N, P, K). Physiologically, combined amendments enhanced photosynthetic performance (chlorophyll, Pn, ΦPSII) and increased activity of key metabolic enzymes (Rubisco, SS, SPS, NR, GS), promoting sucrose, starch, and protein accumulation. LK rapidly raised subsoil pH and potassium levels, ideal for K-deficient orchards. LGOF and LCMA improved overall fertility by supplying Ca and Mg, with LGOF additionally enhancing soil structure in poorly structured acidic soils.

## 1. Introduction

Approximately 40–50% of global potential arable land is classified as acidic (pH < 5.5), with the majority located in tropical and subtropical regions [1]. Human activities, particularly the long-term application of chemical nitrogen fertilizers, have further intensified soil acidification [2,3]. Under acidic conditions, aluminum (Al) toxicity is often considered a primary constraint on plant growth. When soil pH drops below 5.5, Al is solubilized into phytotoxic forms, primarily Al^3+^ and Al(OH)^2+^, which, beyond threshold concentrations, inhibit root elongation, disrupt membrane integrity, and impair water and nutrient uptake [4]. Additionally, low pH per se can impair root development, nutrient acquisition, and photosynthetic performance [5,6,7].

As the highest-yielding fruit crop globally, citrus is susceptible to soil acidification, which may adversely affect its growth [8,9]. The optimal growth pH range for citrus is 5.5–6.5. Exposure to low pH, coupled with high Al availability, leads to significant growth inhibition and reduced longevity [10,11]. Under extreme acidity (pH 2.5), citrus seedlings exhibit severe reductions in biomass accumulation, leaf photosynthetic capacity, and the uptake of macronutrients such as nitrogen (N), phosphorus (P), potassium (K), calcium (Ca), and magnesium (Mg) [8]. Field observations indicate a 13% yield reduction in honey pomelo orchards with soil pH < 4.5 compared to those with pH ≥ 4.5 [12]. Notably, citrus is a deep-rooted crop whose roots can penetrate into the subsoil to acquire water and nutrients [13]. Consequently, subsoil acidification critically limits water and nutrient absorption, ultimately affecting yield and fruit quality. However, current soil amendment practices focus predominantly on the topsoil (0–20 cm) [14,15], while strategies for correcting subsurface acidification in citrus orchards remain largely unexplored.

Conventional amelioration of acidic soils primarily relies on lime application to neutralize acidity and reduce Al solubility [16,17]. However, lime exhibits limited mobility in the soil profile, confining its ameliorative effects mainly to the surface layer [18,19]. Consequently, it often fails to alleviate subsoil acidity and Al toxicity. Long-term lime use may also induce soil compaction and nutrient imbalances [20]. In contrast, organic fertilizers, containing cementing agents such as humic substances and polysaccharides, enhance soil aggregation and improve structural stability [21]. Combined application of lime and organic fertilizer significantly increases exchangeable base cations, cation exchange capacity (CEC), and base saturation compared to lime alone [22]. Similarly, gypsum application provides soluble calcium and facilitates clay-organic matter bridging, thereby improving soil physical properties [23]. Novel amendments such as K_2_CO_3_ and chicken manure ash effectively elevate soil pH. While K_2_CO_3_ is highly soluble and rapidly mobile, chicken manure ash is rich in phosphorus and potassium, contributing to both nutrient supply and pH enhancement.

Integrated soil amendment strategies leveraging the complementary benefits of multiple materials show promise for sustainable acid soil management. However, the synergistic effects and mechanistic actions of lime combined with organic fertilizer, gypsum, K_2_CO_3_, or chicken manure ash remain inadequately studied. We hypothesize that the co-application of lime with one or more of these soil amendments may enhance ameliorative efficiency and could help address the limitations associated with solitary lime application.

Therefore, this study aims to evaluate the effects of lime (L), alone and in combination with gypsum and organic fertilizer (LGOF), K_2_CO_3_ (LK), or chicken manure ash (LCMA), on soil properties and plant performance in ponkan, a major commercial citrus cultivar in subtropical acidic red soil regions of China. A series of parameters, including soil acidity indicators, base cation levels, plant growth performance, root nutrient uptake, and leaf carbon–nitrogen metabolism, were systematically analyzed across different soil layers in a column experiment. Our objectives are to compare the efficacy of different soil amendment combinations in alleviating soil acidity and to elucidate the physiological responses of ponkan seedlings, to help establish a scientific basis for the remediation of subsoil acidification in ponkan production systems.

## 2. Results

### 2.1. Effects of Different Soil Amendments on Soil Acidity

Surface application of the amendments significantly influenced soil pH across different soil layers (Table 1). Compared with the control, L treatment increased the pH of the 0–10 cm and 20–30 cm soil layers while reducing exchangeable hydrogen (H) and exchangeable Al content, but had no significant effect on pH, exchangeable H, or exchangeable Al in soil layers below 20 cm. The LGOF, LK, and LCMA treatments demonstrated a significant ameliorating effect on soil acidity compared to the L treatment. In the 20–30 cm stratum, these treatments exhibited pronounced elevations in pH by 1.24, 1.41, and 1.35 units; substantial reductions in exchangeable H by 35.24%, 41.41%, and 39.21%; and corresponding decreases in exchangeable Al by 40.39%, 38.49%, and 44.84%. This positive trend was also evident in the 30–40 cm stratum, with pH increases of 0.87, 1.04, and 0.94 units; decreases in exchangeable H of 13.89%, 23.81%, and 14.68%; and reductions in exchangeable Al of 21.99%, 32.37%, and 23.05%.

### 2.2. Effects of Different Amendments on Soil Exchangeable Base Cation Content

As shown in Table 2, surface application of soil amendments significantly increased exchangeable K^+^ content in the 20–30 cm and 30–40 cm soil layers, with the LK treatment showing the most pronounced effect. Compared with control, the L treatment increased exchangeable Ca^2+^ content in the 0–10 and 10–20 cm layer but had no significant effect in the 20–30 and 30–40 cm layer. Compared to the L treatment, LGOF and LCMA exhibited a pronounced promoting effect on exchangeable base cations. In the 20–30 cm layer, these treatments led to dramatic increases in exchangeable Ca^2+^ by 452.98% and 135.38%, and significant rises in exchangeable Mg^2+^ by 29.44% and 78.49%. This enhancing effect was also observed in the deeper 30–40 cm layer, where exchangeable Ca^2+^ increased by 128.58% and 45.20%; and exchangeable Mg^2+^ showed significant gains of 9.08% and 17.90%.

### 2.3. Effects of Different Amendments on Soil Acid Buffering Capacity

**Figure 1:**
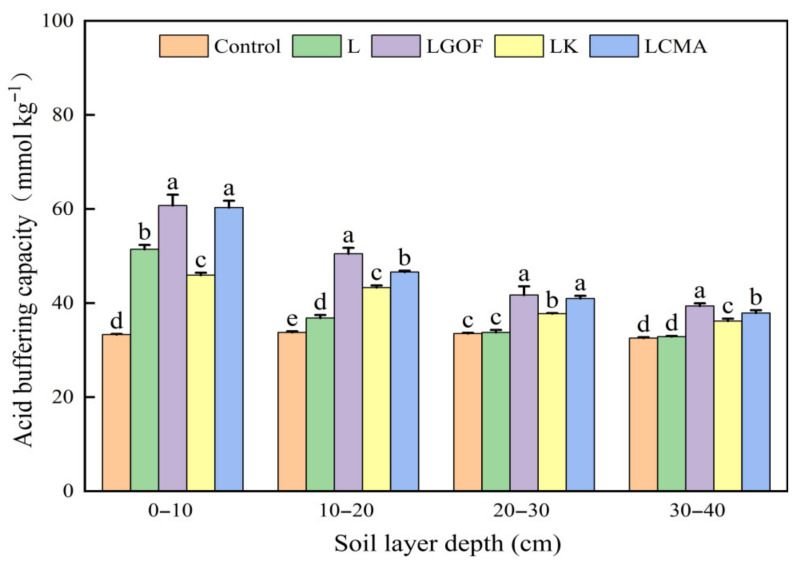
Effects of different soil amendments on the acid buffering capacity across various soil layers. Control, no amendment; L, Lime; LGOF, Lime + Gypsum + Organic fertilizer; LK, Lime + K_2_CO_3_; LCMA, Lime + Chicken manure ash. Values followed by different letters differ significantly among the different treatments (*p* < 0.05); the data are expressed as means ± standard deviation (*n* = 3).

As shown in Figure 1, the L treatment increased the soil acid buffering capacity in the 0–10 cm and 10–20 cm soil layers, but did not significantly affect that in the 20–30 cm and 30–40 cm layers. However, compared to the L treatment, the LK, LGOF, and LCMA treatments exhibited significant increases in the soil acid buffering capacity of the 20–30 cm layer by 23.48%, 11.79%, and 21.35%, respectively. Corresponding increases of 19.84%, 10.04%, and 15.30% were observed in the 30–40 cm layer. These results indicate that lime combined with gypsum and organic fertilizer, lime plus K_2_CO_3_, and lime mixed with chicken manure ash can effectively improve the acid buffering capacity of the deeper soil.

### 2.4. Effects of Different Soil Amendments on Ponkan Seedling Growth

**Figure 2:**
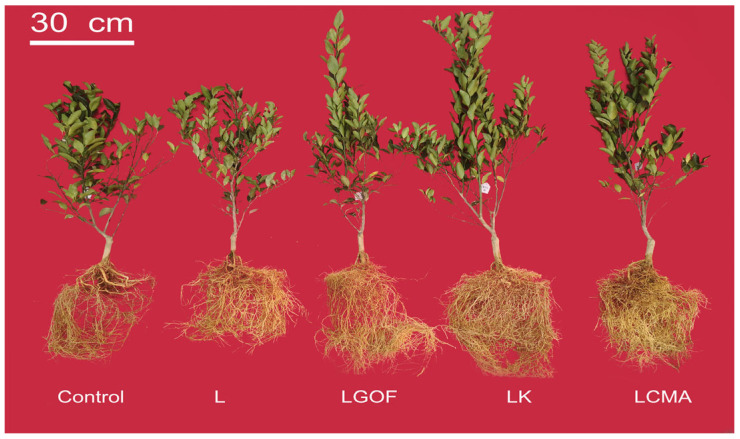
Effects of soil amendments on shoot and root growth of ponkan seedlings. Control, no amendment; L, Lime; LGOF, Lime + Gypsum + Organic fertilizer; LK, Lime + K_2_CO_3_; LCMA, Lime + Chicken manure ash.

**Figure 3:**
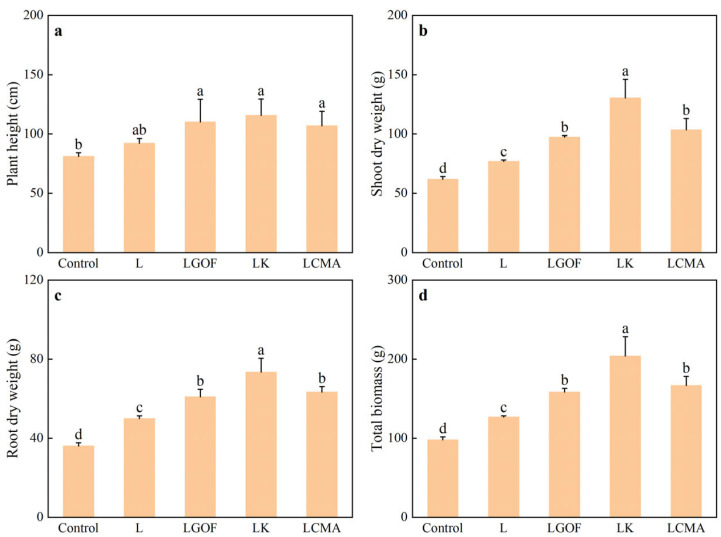
Effects of soil amendments on (**a**) plant height, (**b**) shoot dry weight, (**c**) root dry weight, and (**d**) total biomass of ponkan seedlings. Control, no amendment; L, Lime; LGOF, Lime + Gypsum + Organic fertilizer; LK, Lime + K_2_CO_3_; LCMA, Lime + Chicken manure ash. Values followed by different letters differ significantly among the different treatments (*p* < 0.05); the data are expressed as means ± standard deviation (*n* = 3).

Soil amendments significantly influenced the growth of ponkan seedlings (Figure 2). Seedlings treated with LGOF, LK, and LCMA exhibited better growth than those under control and L treatments, with more numerous and longer root systems. As shown in Figure 3, the L treatment increased root dry weight and total biomass by 38.81% and 29.82%, respectively, compared with the control, though no significant differences were observed in plant height or shoot dry weight. Relative to the L treatment, the LGOF, LK, and LCMA treatments significantly improved plant height, shoot dry weight, root dry weight, and total biomass. Compared with control, lime application significantly increased root length in the 0–10 cm and 10–20 cm layer, root surface area and volume in the 0–10 cm layer, and root tip number in the 10–20 cm layer, but had no significant effect on roots below 20 cm. The LGOF, LK, and LCMA treatments significantly enhanced root length, surface area, volume, and tip number in the 0–10 cm, 10–20 cm, 20–30 cm, and 30–40 cm layers compared with the control (Table S1).

### 2.5. Al Content in Plants

**Figure 4:**
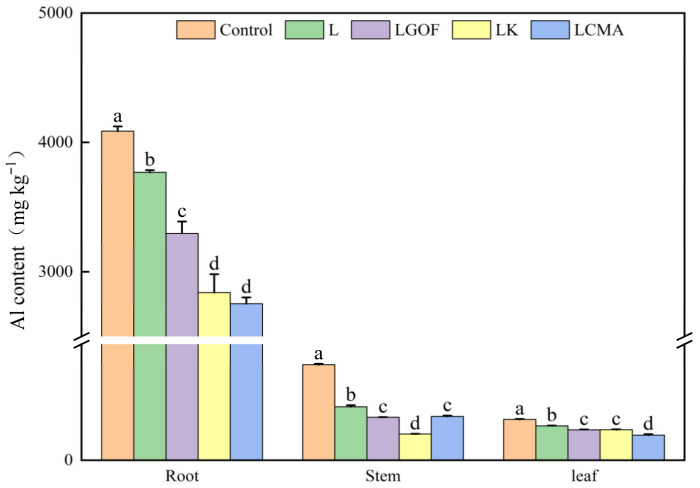
Effects of soil amendments on Al content in different organs of ponkan seedlings. Control, no amendment; L, Lime; LGOF, Lime + Gypsum + Organic fertilizer; LK, Lime + K_2_CO_3_; LCMA, Lime + Chicken manure ash. Values followed by different letters differ significantly among the different treatments (*p* < 0.05); the data are expressed as means ± standard deviation (*n* = 3).

The application of amendments significantly reduced Al content in the roots, stems, and leaves of ponkan seedlings (Figure 4). Compared with the control, the L treatment reduced Al content in root, stems, and leaves by 7.77%, 43.84%, and 15.79%, respectively. Relative to the L treatment, the LGOF, LK, and LCMA treatments further significantly decreased Al content in all plant organs. The above results demonstrate that the soil amendments alleviated Al toxicity in the acidic soil.

### 2.6. Effects of Different Soil Amendments on Nutrient Uptake in Ponkan Seedlings

**Figure 5:**
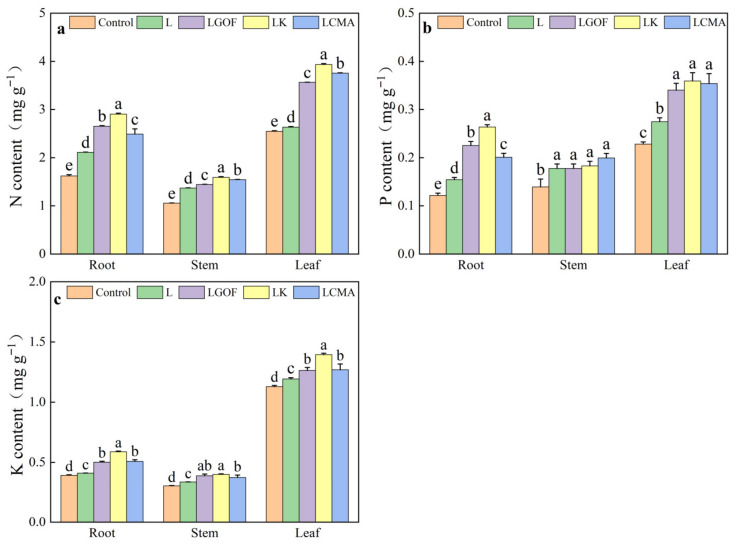
Effects of soil amendments on the (**a**) nitrogen (N) content, (**b**) phosphorus (P) content, and (**c**) potassium (K) content of different organs in ponkan seedling. Control, no amendment; L, Lime; LGOF, Lime + Gypsum + Organic fertilizer; LK, Lime + K_2_CO_3_; LCMA, Lime + Chicken manure ash. Values followed by different letters differ significantly among the different treatments (*p* < 0.05); the data are expressed as means ± standard deviation (*n* = 3).

Soil amendment application significantly increased N, P, and K content in the roots, stems, and leaves of ponkan seedlings (Figure 5). Compared with the control, the L treatment significantly improved N, P, and K content in all plant organs. Compared with the L treatment, LGOF, LK, and LCMA significantly increased N, P, and K content in all plant organs, except that P levels in the stem remained unchanged.

### 2.7. Effects of Different Soil Amendments on Photosynthetic Pigment Content in Ponkan Seedling

**Figure 6:**
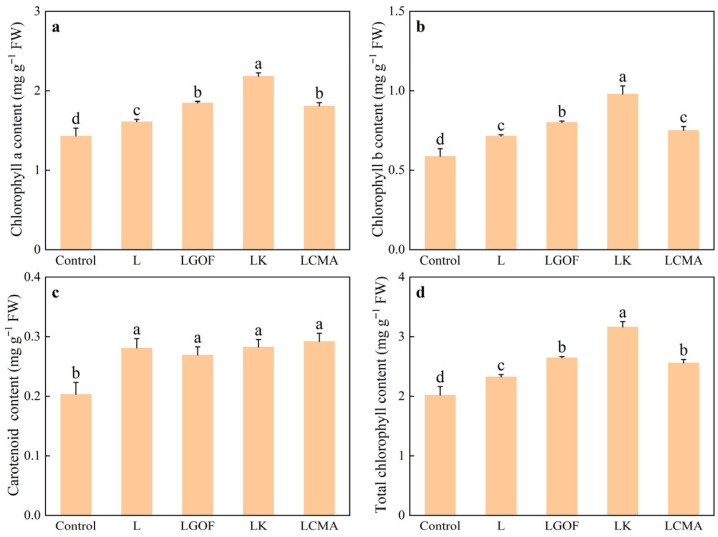
Effect of soil amendments on (**a**) chlorophyll a content, (**b**) chlorophyll b content, (**c**) Carotenoid content, and (**d**) total chlorophyll content in leaves of ponkan seedling. Control, no amendment; L, Lime; LGOF, Lime + Gypsum + Organic fertilizer; LK, Lime + K_2_CO_3_; LCMA, Lime + Chicken manure ash. Values followed by different letters differ significantly among the different treatments (*p* < 0.05); the data are expressed as means ± standard deviation (*n* = 3).

The application of soil amendments significantly increased the content of chlorophyll and carotenoids in the leaves of ponkan (Figure 6). Except for chlorophyll b content in the LCMA treatment, which did not differ significantly from the L treatment, chlorophyll a, chlorophyll b, and total chlorophyll content were significantly higher in the LGOF, LK, and LCMA treatments compared with L, with the LK treatment showing the most notable effect.

### 2.8. Effects of Different Soil Amendments on Chlorophyll Fluorescence Parameters and Photosynthetic Characteristics

**Figure 7:**
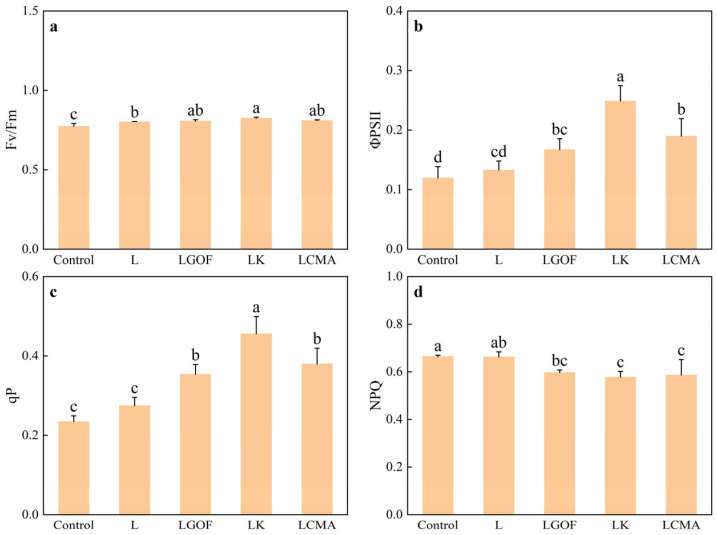
Effect of application of soil amendments on (**a**) maximum photochemical efficiency (Fv/Fm), (**b**) actual photochemical efficiency (ΦPSII), (**c**) photochemical quenching coefficient (qP) and (**d**) non-photochemical quenching coefficient (NPQ) of ponkan. Control, no amendment; L, Lime; LGOF, Lime + Gypsum + Organic fertilizer; LK, Lime + K_2_CO_3_; LCMA, Lime + Chicken manure ash. Values followed by different letters differ significantly among the different treatments (*p* < 0.05); the data are expressed as means ± standard deviation (*n* = 3).

Compared with the control, all amendments increased the maximum photochemical efficiency (Fv/Fm), enhanced the photochemical quenching coefficient (qP), reduced non-photochemical quenching (NPQ), and thereby increased the actual photochemical efficiency (ΦPSII) of ponkan leaves (Figure 7). Relative to the L treatment, the LGOF, LK, and LCMA treatments improved the performance of the PSII reaction center, particularly in light energy capture and conversion efficiency, by 26.02%, 87.48%, and 42.99%, respectively.

**Figure 8:**
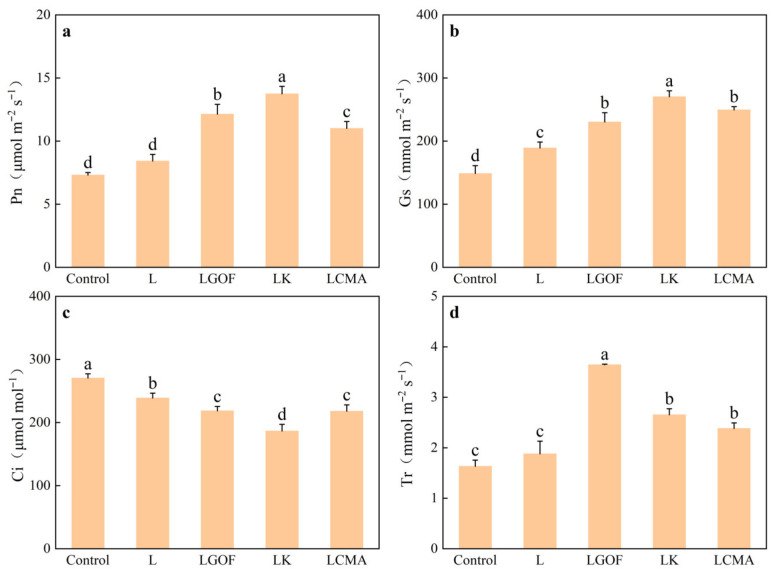
Effects of application of soil amendments on (**a**) net photosynthetic rate (Pn), (**b**) stomatal conductance (Gs), (**c**) intercellular CO_2_ concentration (Ci), and (**d**) transpiration rate (Tr) of ponkan leaves. Control, no amendment; L, Lime; LGOF, Lime + Gypsum + Organic fertilizer; LK, Lime + K_2_CO_3_; LCMA, Lime + Chicken manure ash. Values followed by different letters differ significantly among the different treatments (*p* < 0.05); the data are expressed as means ± standard deviation (*n* = 3).

Soil amendments significantly increased net photosynthetic rate (Pn), stomatal conductance (Gs), and transpiration rate (Tr), while decreasing intercellular CO_2_ concentration (Ci) (Figure 8). Compared with control, the L treatment increased Pn, Gs, and Tr by 15.18%, 27.20%, and 15.34%, respectively, and reduced Ci by 11.67%, with significant differences in Gs and Ci but not in Pn and Tr. Relative to the L treatment, the LGOF, LK, and LCMA treatments further significantly increased Pn, Gs, and Tr, and decreased Ci.

### 2.9. Effects of Different Soil Amendments on Key Enzyme Activities and Product Accumulation in Photosynthesis and Nitrogen Metabolism

**Figure 9:**
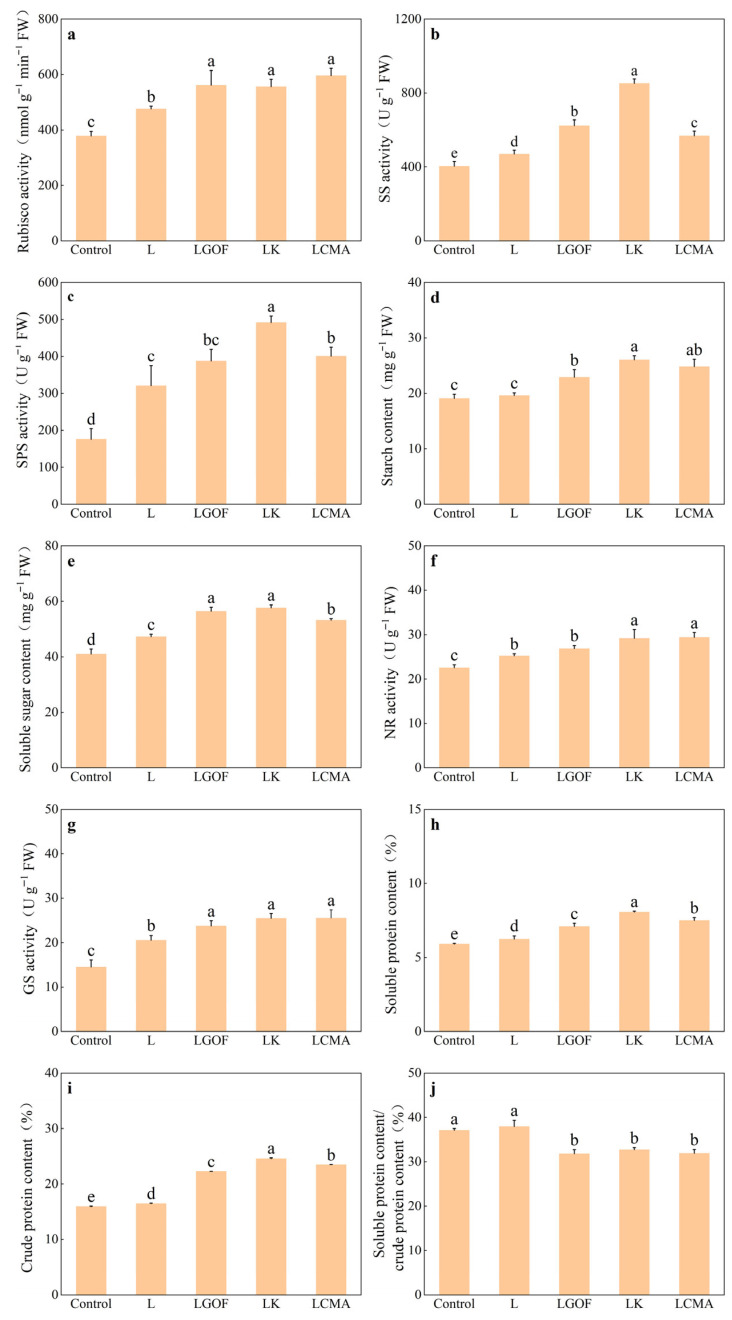
Effect of soil amendments on the (**a**) ribulose-1,5-bisphosphate carboxylase/oxygenase (Rubisco) activity, (**b**) sucrose synthase (SS) activity, (**c**) sucrose phosphate synthase (SPS) activity, (**d**) starch content, (**e**) soluble sugar content, (**f**) nitrate reductase (NR) activity, (**g**) glutamine synthetase (GS) activity, (**h**) soluble protein, (**i**) crude protein, and (**j**) soluble protein/crude protein in ponkan leaves. Control, no amendment; L, Lime; LGOF, Lime + Gypsum + Organic fertilizer; LK, Lime + K_2_CO_3_; LCMA, Lime + Chicken manure ash. Values followed by different letters differ significantly among the different treatments (*p* < 0.05); the data are expressed as means ± standard deviation (*n* = 3).

Soil amendment application significantly enhanced the activities of ribulose-1,5-bisphosphate carboxylase/oxygenase (Rubisco), sucrose synthase (SS), and sucrose phosphate synthase (SPS) in ponkan leaves (Figure 9a–c). Compared with control, the L treatment increased Rubisco, SS, and SPS activities by 25.76%, 16.52%, and 82.25%, respectively. Relative to the L treatment, the LK and LCMA treatments significantly improved the activities of all three enzymes, while the LGOF treatment significantly increased Rubisco and SS activities. Compared to the control, the L treatment increased starch and soluble sugar content by 2.88% and 15.24%, respectively, with the increase in soluble sugar being statistically significant. Furthermore, the L + G + OF, L + K, and L + C treatments further significantly enhanced the leaf starch and soluble sugar content relative to the L treatment. In addition, Soil amendment application significantly enhanced nitrate reductase (NR) and glutamine synthetase (GS) activity, soluble protein and crude protein content in ponkan leaves, with the combined amendments (LGOF, LK, LCMA) showing better effects than lime alone (Figure 9f,g). The ratio of soluble protein to crude protein content reflects protein synthesis capacity; a lower ratio indicates stronger synthesis ability. While the proportion of soluble protein to crude protein showed no significant difference between the L treatment and the control, the LGOF, LK, and LCMA treatments demonstrated significant reductions of 16.12%, 13.68%, and 15.89%, respectively, compared to the control.

## 3. Discussion

### 3.1. Ameliorative Effects of Soil Amendments on Soil Acidification and Al Toxicity

Numerous studies have documented the effects of soil amendments. For instance, traditional lime amendment, due to its low solubility and poor mobility, often ameliorates soil acidity only in the topsoil, failing to address subsoil acidification [18,19]. A long-term wheat-barley-rapeseed rotation experiment demonstrated that annual surface application of lime at 8.5 t ha^−1^ increased soil pH from an initial 5.11 to 6.30 in the 0–10 cm layer and from 4.21 to 5.39 in the 10–20 cm layer, while no significant change was observed in the 20–30 cm layer [18]. In a three-year field study investigating the one-time incorporation of lime at 7.47 t ha^−1^ into the 0–5 cm soil layer in a barley-rapeseed-pea rotation system on sandy loam soil, results showed that compared to the non-limed treatment, lime application increased soil pH by 1.9 units in the 0–5 cm layer and by 0.2 units in the 5–10 cm layer, with no significant change in the 10–20 cm layer [19]. Our results are consistent with these findings, demonstrating that single lime application significantly increased pH only in the 0–10 cm and 10–20 cm layers, with no notable effect observed in the 20–30 cm and 30–40 cm layers. To our knowledge, this study provides the first evidence that the combined amendments LGOF, LK, and LCMA can effectively alleviate subsoil acidification under the specific conditions of a column experiment with ponkan seedlings in a Quaternary red earth-derived Ultisol. These treatments significantly increased the pH and acid buffering capacity while substantially reducing exchangeable H and exchangeable Al contents in the 20–30 cm and 30–40 cm layers. The pronounced improvement from the LGOF treatment likely stems from synergistic mechanisms: (1) The SO_4_^2−^ from gypsum undergoes specific adsorption and ligand exchange with OH^−^ on soil colloids, releasing OH^−^ to neutralize acidity, an effect known as “self-liming” [24]. (2) SO_4_^2−^ can also form neutral ion pairs such as CaSO_4_^0^ with Ca^2+^ [25]. These uncharged complexes are highly mobile in the soil profile. During migration, CaSO_4_^0^ dissociates, releasing Ca^2+^, which replaces exchangeable Al and H on colloids, thereby neutralizing acidity and mitigating Al toxicity in deeper layers. (3) The organic fertilizer contributes through its rich humus content; its functional groups, such as carboxyl and phenolic hydroxyl, form stable organo-aluminum complexes, which reduce active Al toxicity and inhibit the hydrolysis of Al^3+^ that generates H^+^ [26]. Concurrently, organic fertilizer releases alkaline cations that consume exchangeable acidity, leading to a pH increase [27]. Both LK and LCMA treatments increased subsoil base-cation concentrations and reduced acidity. This effect is presumably because K_2_CO_3_ and chicken-manure ash rapidly release K^+^, while their strong alkalinity neutralizes acidity, diminishing H^+^ and Al^3+^ competition for exchange sites [28]. Furthermore, both treatments increase soil pH, thereby enhancing the nitrification of ammonium (NH_4_^+^) to nitrate (NO_3_^−^). As a negatively charged ion, NO_3_^−^ is poorly adsorbed by soil colloids and is prone to leaching. This leaching process necessitates charge-balancing co-transport with base cations such as K^+^, Ca^2+^, and Mg^2+^ [29], thereby facilitating the movement of these nutrients into deeper soil layers. The assertion that intensified nitrification drives the co-transport of cations with NO_3_^−^ to deeper layers is solely a mechanistic hypothesis that still requires verification through determinations of nitrogen forms and leaching profiles. In addition to mitigating soil acidity, the combined amendments significantly improved the balance of base cations (Table 2). Specifically, the LGOF, LK, and LCMA treatments markedly increased the concentrations of exchangeable K^+^, Ca^2+^, and Mg^2+^. This enhancement helped alleviate potential nutrient antagonism—such as competition between Ca^2+^ and K^+^ or Mg^2+^, often induced by long-term sole lime application. As a result, these amendments contributed to a more harmonized soil nutrient environment conducive to plant growth. It should be noted that this alleviation of cation antagonism is inferred from soil-exchangeable data alone; tissue-based confirmation (e.g., leaf or root Ca, Mg content) is required to substantiate the beneficial effect on plant nutrition.

### 3.2. Effects of Soil Amendments on Alleviating Aluminum Toxicity and Improving Root Architecture and Nutrient Uptake in Ponkan

The efficacy of soil amendments in alleviating soil acidification, reducing Al accumulation in plants, and promoting plant development has been well documented in previous studies [30]. Consistent with these findings, our research demonstrates that the application of combined amendments significantly increased soil pH and markedly decreased aluminum content in the roots, stems, and leaves of ponkan trees. Concurrently, substantial improvements were observed in root morphological parameters, including root length, surface area, volume, and tip number, as well as in above-ground biomass. These results align with observations in pomelo, where optimized NPK fertilization combined with lime application raised soil pH by 1.25 units, reduced root aluminum concentration by 47.8%, and increased root length and root tip number by 80.3% and 136.6%, respectively [15]. Under low pH conditions, the uptake of essential nutrients such as N, P, and K is impaired, as previously documented in eggplant [31] and citrus [32]. In this study, the application of combined amendments significantly increased the N, P, and K concentrations in the roots, stems, and leaves of ponkan (Figure 5). This improvement may be attributed to: (1) increased root volume and absorption surface area, which enhanced the plant’s capacity to explore and acquire soil nutrients; and (2) a comprehensive mitigation of the multiple inhibitory effects of soil acidification on nutrient uptake. Specifically, soil acidification impedes the conversion of ammonium nitrogen to nitrate nitrogen [33], thereby reducing nitrate availability and uptake by plants. Additionally, soil acidification mobilizes iron (Fe) and Al, enhancing the formation of insoluble Fe- and Al-phosphates that reduce P availability and limit plant P uptake [34,35]. Furthermore, in acidic environments, Al^3+^ competes with K^+^ for root absorption sites, thereby suppressing potassium acquisition [36].

### 3.3. Synergistic Enhancement of Photosynthetic Characteristics and Carbon–Nitrogen Metabolism by Soil Amendments

Low soil pH, per se, can damage the oxygen-evolving complex (OEC) and electron transport chain of PSII in citrus leaves. Furthermore, active Al^3+^ exacerbates oxidative stress and ionic imbalance, leading to damage at the PSII reaction centers and impeding photosynthetic electron transport [8,15,37]. Our results showed that all amendment treatments increased chlorophyll content (Figure 6). Amendments enhanced Fv/Fm, increased qP, and decreased NPQ, consequently raising the effective ΦPSII. The combined treatments (LGOF, LK, LCMA) consistently outperformed the sole lime treatment. This suggests that by neutralizing soil acidity, the amendments removed the fundamental stressor on the photosynthetic apparatus, facilitating normal chlorophyll synthesis and the recovery of photosynthetic function in ponkan.

The significant increase in Pn (Figure 8a) represents the ultimate manifestation of enhanced photosynthetic assimilation in ponkan. Although Gs increased under all soil amendment treatments, the significant decrease in Ci indicates that the rise in Pn was primarily governed by non-stomatal factors, i.e., an improvement in the mesophyll cells’ intrinsic photosynthetic capacity. Under pH 2.5 acidification stress, the abundance of proteins in citrus related to Rubisco activation, the Calvin cycle, electron transport, and chloroplast development, including Rubisco activase (RCA), Rubisco large subunit-binding protein (Cpn60 β), FNR, TROL, PGK1, and MECT, was significantly altered. These changes led to decreased chlorophyll content, electron transport rate (ETR), and net photosynthetic rate, while excessive starch accumulation further exacerbated structural damage to the chloroplasts [38]. Rubisco, the rate-limiting enzyme of the Calvin cycle, directly determines carbon assimilation rate [39]. Consistent with findings in soybean [40], the combined amendments significantly enhanced Rubisco activity compared to the sole lime treatment (Figure 9a). Concurrently, increased activities of SS and SPS promoted the conversion and transport of photoassimilates into sucrose. This prevented the feedback inhibition of photosynthesis caused by excessive carbohydrate accumulation in leaves, thereby maintaining the efficient operation of the photosynthetic machinery. These combined effects led to significant accumulation of soluble sugars and starch in the leaves (Figure 9d,e). These abundant photosynthetic products are not only the fundamental source for increased plant biomass but also direct precursors for sugar accumulation in fruits, laying the material foundation for potential future improvements in fruit quality.

NR and GS are key enzymes in plant nitrogen assimilation, and their activities reflect the plant’s capacity to metabolize inorganic nitrogen [41,42]. Consistent with this, our study found that the combined amendment treatments significantly increased the activities of NR and GS in the leaves (Figure 9f,g), thereby promoting efficient nitrogen assimilation. Notably, while crude protein content increased significantly, the ratio of soluble protein to crude protein decreased. This indicates that nitrogen was preferentially allocated towards the synthesis of structural and functional proteins rather than being retained in soluble storage or stress-related forms, suggesting a metabolic shift from defense to growth. The synchronized enhancement of key enzymes involved in leaf C and N metabolism, coupled with the significant accumulation of photosynthetic products, ensured the supply of carbon skeletons and energy, achieving a synergistic enhancement of carbon and nitrogen metabolism. This synergy constitutes the core physiological basis for the observed biomass accumulation in the ponkan seedlings.

In this study, each soil column was treated as an independent experimental unit. Treatment effects were therefore compared separately by depth to assess differences between treatments within each soil layer. Owing to the limited number of replicates, a split-plot or mixed-effects model incorporating “depth” as a factor and “pot” as a random effect was not employed. Consequently, the “treatment × depth” interaction could not be fully tested, which may affect the interpretation of treatment effect patterns with depth. This limitation is inherent to such exploratory research. Future studies should increase replication and adopt more comprehensive models to better reveal the integrated effects of treatments throughout the soil profile.

## 4. Materials and Methods

### 4.1. Experimental Site and Materials

The experiment was conducted from March to December 2024 at the field experiment base of the College of Horticulture, Fujian Agriculture and Forestry University (119°23′ E, 26°08′ N), located in Fuzhou City, Fujian Province, China. The site experiences a subtropical monsoon climate with a mean annual temperature of 19.8 °C and a mean annual precipitation of 1390 mm. The soil was an Ultisol derived from Quaternary red earth, collected from the Tianma Citrus Orchard in Yongchun County, Quanzhou City, Fujian Province, China. The physicochemical properties of the soil were as follows: pH 3.88, organic matter 14.45 g kg^−1^, available nitrogen 71.75 mg kg^−1^, available phosphorus 22.57 mg kg^−1^, available potassium 59.63 mg kg^−1^, exchangeable calcium 162.25 mg kg^−1^, exchangeable magnesium 35.75 mg kg^−1^, available sulfur 134.3 mg kg^−1^, exchangeable H 1.9 cmol kg^−1^, exchangeable Al 2.55 cmol kg^−1^.

The test plants were current-year, uniformly growing ponkan (*Citrus reticulata* Blanco cv. Ponkan) seedlings grafted onto trifoliate orange (*Poncirus trifoliata*) rootstock, provided by the Yongchun Lvyuan Citrus Seedling Nursery. The amendments lime, gypsum, and K_2_CO_3_ were chemical pure reagents. The organic fertilizer (OF), provided by Fujian Zhongke Huasheng Agricultural Development Co., Ltd. (Fuzhou, China), was primarily produced using mushroom residue as the raw material. Its main properties were: pH 6.70, organic matter 39.70%, N 2.38%, P_2_O_5_ 2.39%, K_2_O 1.79%. Chicken manure ash (CMA), provided by Sanming Beisinuo Agricultural Technology Co., Ltd., (Sanming, China) was an ash residue from a thermal power plant using a mixture of chicken manure and rice hulls as fuel. Its main properties were: pH 12.37, P_2_O_5_ 10.42%, K_2_O 9.10%, Ca 3.86%, Mg 2.70%.

### 4.2. Experimental Design

A column experiment was conducted using PVC pipes with an inner diameter of 30 cm and a height of 50 cm. Soil was packed layer by layer from the bottom up according to a bulk density of 1.20 g cm^−3^. The soil height in each PVC pipe was 40 cm, with a soil weight of 33.91 kg. The experiment was set up with five treatments, each defined as: no soil amendment (Control), lime alone (L), lime in combination with gypsum and organic fertilizer (LGOF), lime with K_2_CO_3_ (LK), and lime mixed with chicken manure ash (LCMA). Each treatment was replicated three times.

Prior to the experiment, incubation tests were conducted to determine the amendment rates required to adjust the soil pH to 6.0: lime 3.38 g kg^−1^, K_2_CO_3_ 3.10 g kg^−1^, chicken manure ash 18.05 g kg^−1^. Consequently, the lime application rates for the L and LGOF treatments were both 3.38 g kg^−1^. The lime and K_2_CO_3_ application rates for the LK treatment were 1.69 g kg^−1^ and 1.55 g kg^−1^, respectively. The lime and chicken manure ash application rates for the LCMA treatment were 1.69 g kg^−1^ and 9.03 g kg^−1^, respectively. The application rates of gypsum and organic fertilizer were based on field management practices, set at 3.57 g kg^−1^ and 42.86 g kg^−1^, respectively. The amounts of N, P, and K nutrients introduced by the organic fertilizer, K_2_CO_3_, and chicken manure ash were balanced across all treatments by adding urea (N 46.67%), ammonium phosphate (diammonium phosphate, N 21.20%, P_2_O_5_ 53.76%), and potassium sulfate (K_2_O 54.16%).

On 7 March 2024, the first 25% of the total amendment dose was thoroughly mixed into the 0–10 cm soil layer along with standardized fertilizers to equalize nutrient inputs across all treatments. Each soil column was then planted with a single ponkan seedling, with the root system confined to the 0–15 cm depth during planting. The remaining 75% of the amendments, supplemented with balancing fertilizers, were divided into three equal portions and incorporated into the 0–10 cm layer on 19 May, 24 July, and 1 September, respectively. The column experiment was conducted under outdoor conditions, with soil moisture consistently maintained across all treatments using the weighing method. The columns were arranged in a completely randomized design within a 5 × 3 grid. To minimize edge effects and micro-environmental variations, the columns were completely re-randomized every 15 days. Tap water (pH 6.24) was used for irrigation. Soil moisture was monitored by weighing each PVC column every two days. When the weight fell below the threshold corresponding to 80% of the maximum field water capacity, deionized water was added in the evening to restore moisture to the target level. If natural rainfall raised soil moisture above the threshold, irrigation was suspended for all treatments until the moisture content again dropped below the threshold. All PVC columns were equipped with bottom drainage holes to prevent waterlogging. This approach ensured consistent moisture conditions across all treatments throughout the growth period.

### 4.3. Determination of Soil Acidity and Exchangeable Base Cation Content

In December 2024, the PVC pipes were cut open from the side. Soil samples were collected, avoiding plant roots at 10 cm intervals from top to bottom. Each soil sample was freed of impurities, passed through a 1 mm sieve, and air-dried. Soil pH was measured potentiometrically using a pH meter (soil:water ratio 1:2.5). Exchangeable H and Exchangeable Al were quantified via KCl extraction followed by NaOH neutralization titration. The soil acid buffering capacity was assessed by titration after extraction with CO_2_-free distilled water [26,43]. Exchangeable K^+^ was measured by flame photometry (PerkinElmer PinAAcle 900T, Waltham, MA, USA). Exchangeable Ca^2+^ and Mg^2+^ contents were determined using inductively coupled plasma optical emission spectrometry (ICP-OES; PerkinElmer Avio 200, Waltham, MA, USA).

### 4.4. Determination of Plant Growth Indicators

Before opening the PVC pipes, the plant height was recorded. After soil collection, roots were washed free of soil using a water gun. Root length, root surface area, number of root tips, and root diameter at different soil depths were measured using a root scanner (WinRhizo Pro LA2400, Regent Instruments, Québec, QC, Canada). Plants were divided into roots, stems, and leaves. These parts were placed in an oven at 105 °C for 30 min for enzyme deactivation and then dried at 80 °C to constant weight. The dry weights of roots, stems, and leaves were recorded, and the total biomass (root dry weight + stem dry weight + leaf dry weight) was calculated.

### 4.5. Determination of N, P, K, and Al Contents in Ponkan Seedlings

The dried roots, stems, and leaves were ground and passed through a 100-mesh sieve. The sieved samples were digested with H_2_SO_4_–H_2_O_2_. N content was determined using a continuous flow analyzer (SKALAR San++, Skalar Analytical B.V., Breda, Netherlands). P content was determined by the vanadomolybdate yellow colorimetric method [44]. K content was determined by flame photometry (PerkinElmer PinAAcle 900T, Waltham, MA, USA). Al content was determined by inductively coupled plasma optical emission spectrometry (ICP-OES; iCAP 7400, Thermo Fisher Scientific, Waltham, MA, USA) after HNO_3_–HClO_4_ digestion of plant tissue samples.

### 4.6. Photosynthetic Pigment Content

Following an 8-month growth period (7 November 2024), the fully expanded 3rd to 6th leaves from each treatment were sampled at 9:00 AM. The collected samples were immediately flash-frozen in liquid nitrogen and subsequently stored at −80 °C under dark conditions. Chlorophyll and carotenoid contents were extracted using an 80% (*v*/*v*) acetone aqueous solution in the dark and measured by a spectrophotometer [45,46].

### 4.7. Chlorophyll Fluorescence Parameters and Photosynthesis Characteristics

After 8 months of plant growth (11 November 2024), the first leaf from the second branch from the top of the plant was selected at 9:00 AM, dark-adapted for 30 min, and then measured using a chlorophyll fluorescence imaging system (IMAGING-PAM, Heinz Walz GmbH, Effeltrich, Germany) to determine the Fv/Fm, qP, NPQ, and ΦPSII. The 1st, 2nd, and 3rd leaves from the top of the first branch from the top of the plant were selected, avoiding the main veins. Under standardized conditions of approximately 400 ± 2 µmol mol^−1^ CO_2_ and a light intensity of 1000 µmol m^−2^ s^−1^ PPFD. The leaf temperature was maintained at 26.1 ± 0.3 °C with a relative humidity of 60 ± 3%. Key gas exchange parameters, including Pn, Ci, Gs, and Tr, were measured. Measurements were taken on sunny days between 9:00 and 11:00 AM using a CIRAS-4 portable photosynthesis system (PP Systems, Amesbury, MA, USA). For each plant, the first to third leaves from the first branch were selected. For each leaf, measurements were taken three times as technical replicates and averaged. The average values from the three leaves were then combined to represent the plant value. Each treatment included three plants, which served as biological replicates (*n* = 3). Results are expressed as mean ± standard deviation.

### 4.8. Determination of Carbon and Nitrogen Metabolism-Related Enzyme Activities and Product Content in Ponkan Seedlings

Leaves used for gas exchange parameter measurements were cut and stored in liquid nitrogen for subsequent biochemical analysis. The activities of Rubisco, SS, SPS, NR, and GS were determined using assay kits (Solarbio, Beijing, China). Soluble protein, soluble sugar and starch contents were determined using kits from Suzhou Comin Biotechnology Co., Ltd. (Cominbio, Suzhou, China). Dried leaf samples were ground to pass a 100-mesh sieve and digested with H_2_SO_4_–H_2_O_2_. The crude protein content was subsequently quantified by continuous-flow analysis. All analyses were performed in triplicate, and the results are presented as means.

### 4.9. Data Analysis

Data were processed using Microsoft Excel 2010 (Microsoft Inc., Redmond, WA, USA) to calculate means and standard deviations. Statistical analyses were performed with SPSS 26.0 (International Business Machines Corporation, New York, NY, USA). Data normality and homogeneity of variances were assessed using the Shapiro–Wilk test and Levene’s test, respectively. Upon confirming homogeneity of variances, a one-way analysis of variance (ANOVA) was conducted, followed by Duncan’s multiple range test to compare differences among treatment means at a significance level of *p* < 0.05. All graphs were generated using Origin 2021 (OriginLab Corp., Northampton, MA, USA), with error bars representing ± standard deviation (SD).

## 5. Conclusions

The present study demonstrates that the combined amendments (LGOF, LK, and LCMA) were more effective than lime alone in alleviating subsoil acidification, enhancing soil acid buffering capacity and base cation content, and promoting the growth as well as nitrogen, phosphorus, and potassium uptake of ponkan seedlings. Compared to the lime-alone treatment, the combined amendments improved the photosynthetic rate, up-regulated the activities of key carbon metabolism enzymes (e.g., Rubisco, invertase, sucrose phosphate synthase) and nitrogen metabolism enzymes (e.g., nitrate reductase, glutamine synthetase), and facilitated the accumulation of sucrose, starch, and protein in the seedlings. Among these amendments, the LK treatment stood out for its rapid effectiveness in raising the pH of the deeper soil layers. The high solubility and mobility of potassium carbonate enabled it to migrate downward quickly, neutralizing subsoil acidity while significantly increasing the exchangeable potassium content. This makes LK a potentially optimal choice for orchards suffering from severe potassium deficiency and pronounced subsoil acidification. In contrast, the LGOF and LCMA treatments offered superior advantages in enhancing overall soil fertility. They not only effectively ameliorated acidity in the middle and lower soil profiles but also markedly increased the levels of exchangeable calcium, magnesium. The incorporation of organic fertilizer in the LGOF treatment further improved soil structure and promoted aggregate formation. Therefore, we recommend the following tailored amendments. For acidic soils with severe potassium deficiency, LK is advised, whereas for those with overall poor fertility, either LGOF or LCMA should be used, as they provide integrated improvement by addressing both acidity and multiple nutrient deficiencies.

This work provides a systematic pilot report on strategies for ameliorating subsoil acidification in acidic soils under ponkan cultivation, offering important theoretical insights and a technical framework for addressing these practical challenges. As vertical water flow and ion migration in the soil columns may differ from field conditions, the magnitude of the observed effects could be smaller in mature orchards. Moreover, this study was confined to column experiments using ponkan seedlings. These controlled conditions cannot fully replicate the complexities of a mature orchard ecosystem, and the short duration of the trial precluded an assessment of the long-term effectiveness of the amendments. Therefore, future research should validate these findings in field trials with fruit-bearing ponkan trees, with a focus on the persistence of the amendments, tree yield, and fruit quality. This work provides a systematic pilot report on strategies for ameliorating subsoil acidification in acidic soils under ponkan cultivation, offering important theoretical insights and a technical framework for addressing these practical challenges. As vertical water flow and ion migration in the soil columns may differ from field conditions, the magnitude of the observed effects could be smaller in mature orchards. Moreover, this study was confined to column experiments using ponkan seedlings. These controlled conditions cannot fully replicate the complexities of a mature orchard ecosystem, and the short duration of the trial precluded an assessment of the long-term effectiveness of the amendments. Therefore, future research should validate these findings in field trials with fruit-bearing ponkan trees, with a focus on the persistence of the amendments, tree yield, and fruit quality.

## Figures and Tables

**Table 1:** Effects of soil amendments on the acidity of different soil layers.

Soil Depth (cm)	**Treatment**	**pH**	**Exchangeable H (cmol kg** **^−^** **^1^** **)**	**Exchangeable Al (cmol kg** **^−^** **^1^** **)**
0–10	Control	3.80 ± 0.03 d	1.77 ± 0.06 a	2.35 ± 0.10 a
	L	7.76 ± 0.06 a	0.12 ± 0.03 b	0.08 ± 0.03 b
	LGOF	7.56 ± 0.07 b	0.12 ± 0.03 b	0.07 ± 0.03 b
	LK	7.21 ± 0.11 c	0.13 ± 0.03 b	0.12 ± 0.03 b
	LCMA	7.45 ± 0.13 b	0.13 ± 0.03 b	0.08 ± 0.03 b
10–20	Control	3.74 ± 0.01 d	2.02 ± 0.08 a	2.58 ± 0.06 a
	L	5.02 ± 0.04 c	1.12 ± 0.03 b	1.32 ± 0.08 b
	LGOF	6.25 ± 0.03 a	0.52 ± 0.06 c	0.29 ± 0.05 e
	LK	6.11 ± 0.02 b	0.48 ± 0.03 c	0.72 ± 0.03 c
	LCMA	6.17 ± 0.09 ab	0.47 ± 0.03 c	0.47 ± 0.03 d
20–30	Control	3.65 ± 0.02 c	2.27 ± 0.06 a	2.52 ± 0.10 a
	L	3.70 ± 0.04 c	2.27 ± 0.03 a	2.55 ± 0.10 a
	LGOF	4.94 ± 0.11 b	1.47 ± 0.06 b	1.52 ± 0.08 b
	LK	5.11 ± 0.07 a	1.33 ± 0.06 c	1.55 ± 0.09 b
	LCMA	5.05 ± 0.10 ab	1.38 ± 0.03 bc	1.42 ± 0.08 b
30–40	Control	3.57 ± 0.01 d	2.58 ± 0.10 a	2.90 ± 0.15 a
	L	3.58 ± 0.02 d	2.52 ± 0.07 a	2.82 ± 0.08 a
	LGOF	4.45 ± 0.03 c	2.17 ± 0.08 b	2.20 ± 0.05 b
	LK	4.62 ± 0.01 a	1.92 ± 0.03 c	1.97 ± 0.06 c
	LCMA	4.52 ± 0.04 b	2.15 ± 0.05 b	2.17 ± 0.08 b

Control, no amendment; L, Lime; LGOF, Lime + Gypsum + Organic fertilizer; LK, Lime + K_2_CO_3_; LCMA, Lime + Chicken manure ash. Values followed by different letters differ significantly among the different treatments (*p* < 0.05); the data are expressed as means ± standard deviation (*n* = 3).

**Table 2:** Effects of soil amendments on the content of base ions in different soil layers.

**Soil Depth (cm)**	**Treatment**	**Exchangeable K^+^**(mg kg^−1^)	**Exchangeable Ca^2+^** **(mg kg** **^−^** **^1^** **)**	**Exchangeable Mg^2+^** **(mg kg** **^−^** **^1^** **)**
0–10	Control	961.35 ± 20.30 a	152.07 ± 11.74 e	34.90 ± 0.92 c
	L	986.11 ± 58.69 a	2520.79 ± 71.10 b	33.32 ± 0.56 c
	LGOF	712.24 ± 16.15 b	3511.99 ± 173.87 a	179.04 ± 6.87 b
	LK	527.21 ± 24.07 d	1004.56 ± 44.77 d	35.73 ± 0.28 c
	LCMA	630.13 ± 15.72 c	1538.99 ± 40.73 c	286.37 ± 20.79 a
10–20	Control	587.45 ± 11.91 b	167.85 ± 4.00 e	37.78 ± 1.34 c
	L	584.23 ± 60.24 b	619.53 ± 37.77 c	32.01 ± 7.38 c
	LGOF	666.06 ± 43.98 a	1930.62 ± 134.75 a	72.43 ± 1.81 b
	LK	710.59 ± 30.43 a	379.33 ± 11.08 d	36.30 ± 0.54 c
	LCMA	644.79 ± 15.31 ab	827.50 ± 23.87 b	104.36 ± 3.22 a
20–30	Control	381.17 ± 73.00 c	163.59 ± 11.12 c	35.24 ± 0.59 c
	L	348.95 ± 72.34 c	163.03 ± 3.31 c	34.82 ± 0.87 c
	LGOF	509.00 ± 57.69 ab	901.52 ± 14.87 a	45.07 ± 1.36 b
	LK	523.37 ± 56.57 a	150.78 ± 7.19 c	34.73 ± 0.52 c
	LCMA	460.61 ± 60.61 b	383.74 ± 19.05 b	62.15 ± 3.35 a
30–40	Control	286.66 ± 29.86 d	154.99 ± 2.32 c	35.61 ± 0.46 c
	L	277.15 ± 11.27 d	155.12 ± 8.72 c	34.69 ± 0.64 c
	LGOF	352.39 ± 11.35 c	354.58 ± 15.17 a	37.84 ± 0.53 b
	LK	500.35 ± 20.51 a	142.80 ± 3.99 c	35.05 ± 0.17 c
	LCMA	416.22 ± 17.39 b	225.23 ± 22.61 b	40.90 ± 0.45 a

Control, no amendment; L, Lime; LGOF, Lime + Gypsum + Organic fertilizer; LK, Lime + K_2_CO_3_; LCMA, Lime + Chicken manure ash. Values followed by different letters differ significantly among the different treatments (*p* < 0.05); the data are expressed as means ± standard deviation (*n* = 3).

## Data Availability

Data are contained within the article. The data presented in this study can be requested from the authors.

## Supplementary Materials

The following supporting information can be downloaded at: https://www.mdpi.com/article/10.3390/plants14233613/s1, Table S1: Effects of different amendments on morphological indices of ponkan seedlings root in soil layers.
