# Supplementary material for: Integrated Soil Amendments Alleviate Subsoil Acidification and Enhance Ponkan Seedling Growth in a Column Experiment
Source: Plants (Basel). 2025 Nov 26;14(23):3613. doi: 10.3390/plants14233613 (PMC12694400; doi:10.3390/plants14233613)
Supplement: Supplementary file 1 [file plants-14-03613-s001.zip › plants-3985569-supplementary.pdf]

plants

MDPI

Supplementary Materials

Table S1. Effects of different amendments on morphological indices of ponkan seedlings root in soil layers.

Soil depth (cm)	Treatment	Root length (cm)	Root surface area（cm²）	Root volume（cm³）	Root tip number
0-10	Control	1026.64 ± 234.27 b	413.52 ± 56.75 b	10.2 1± 0.61 c	3639.67± 572.76 b
	L	2289.64 ± 238.32 a	558.47 ± 48.67 a	14.91 ± 0.40 b	3784.33 ± 727.56 b
	LGOF	2323.68 ± 141.88 a	545.73 ± 3.34 a	17.39 ± 1.38 ab	4951.00 ± 569.79 ab
	LK	2974.61 ± 827.79 a	670.66 ± 112.22 a	21.31 ± 3.95 a	9870.00 ± 4603.21 a
	LCMA	2307.85 ± 26.14 a	570.60 ± 64.10a	17.00 ± 1.43 b	5018.67 ± 637.69 ab
10-20	Control	1824.15 ± 142.22 c	713.64 ± 75.51 c	22.24 ± 3.07 c	4838.00 ± 447.71 c
	L	5514.39 ± 680.27 b	1120.29 ± 98.76 bc	28.58 ± 4.27 c	10665.67 ± 392.57 b
	LGOF	4763.84 ± 202.96 b	1368.18 ± 204.72 b	42.30 ± 3.60 b	10585.67 ± 408.57 b
	LK	9472.90 ± 3131.45 a	2330.78 ± 645.94 a	56.71 ± 9.73 a	29537.00 ± 6045.72 a
	LCMA	5249.64 ± 585.64 b	1666.43 ± 160.87 b	44.59 ± 4.52 b	11244.67 ± 1184.70 b
20-30	Control	2445.83 ± 361.06 b	754.50 ± 155.63 b	18.59 ± 2.42 c	4806.67 ± 930.09 c
	L	3038.42 ± 314.92 b	817.47 ± 171.55 b	20.57 ± 2.05 bc	4228.33 ± 832.87 c
	LGOF	4313.33 ± 745.14 a	1165.75 ± 152.06 a	23.97 ± 1.89 b	7796.33 ± 508.10 b
	LK	4635.17 ± 947.30 a	1189.83 ± 84.63 a	28.05 ± 2.21 a	13605.00 ± 2844.32 a
	LCMA	4423.81 ± 436.13 a	1251.10 ± 168.09 a	31.43 ± 2.32 a	7877.33 ± 823.21 b
30-40	Control	1921.25 ± 374.17 c	582.78 ± 211.96 b	13.66 ± 3.35 c	3881.67 ± 715.66 b
	L	2229.25 ± 192.79 c	555.70 ± 146.05 b	13.28 ± 4.53 c	3277.33± 783.58 b
	LGOF	3544.83 ± 93.43 ab	951.75 ± 34.32 a	19.49 ± 0.80 b	6222.00 ± 1646.38 ab
	LK	4280.45 ± 970.70 a	1120.62 ± 84.00 a	24.98 ± 0.93 a	10562.00 ± 4814.86 a
	LCMA	3266.75 ±303.08 b	896.58 ± 38.06 a	19.87 ± 1.42 b	6020.0 ± 1669.98 ab

Control, no amendment; L, Lime; LGOF, Lime + Gypsum + Organic fertilizer; LK, Lime + K_2CO_3; LCMA, Lime + Chicken manure ash. Values followed by different letters differ significantly among the different treatments ($p < 0.05$); the data are expressed as means ± standard deviation ($n = 3$).
